# Delaying early morning workouts to protect sleep in two-a-day athletes

**DOI:** 10.3389/fphys.2024.1346761

**Published:** 2024-01-16

**Authors:** Shawn D. Youngstedt

**Affiliations:** Edson College of Nursing and Health Innovation, Arizona State University, Phoenix, AZ, United States

**Keywords:** performance, circadian, night owl, sleep loss, depression

## Abstract

Two-a-day training is common for endurance athletes with training sessions typically beginning at 6 a.m. and 3 p.m. or 4 p.m. However, the early morning workouts could contribute to significant sleep loss, especially for night owls. Chronic sleep loss over a season could result in impaired performance, as well as an increased risk of physical and mental illness. It is hypothesized that shifting the early morning workout to later in the day could have beneficial effects for these athletes. A number of obstacles could make this hypothesis difficulty to test and implement. However, such a change could have dramatic benefits for some athletes.

## Introduction

Two-a-day training is a time-honored tradition for endurance athletes (Tønnessen et al., 2014), with training sessions typically starting at 6 a.m. and 3–4 p.m. Two-a-day training is justified by research and coaching experience as a means of achieving a greater total volume of training (e.g., meters of swimming) and a greater amount of high intensity training than training once per day (Haugen et al., 2022).

However, waking up and preparing for a 6 a.m. practice is liable to involve a significant loss of sleep for many athletes (Samuels, 2008), particularly for extreme “night owls” who are unable to go to sleep before 2 or 3 a.m. Chronic loss of sleep over one or more seasons could have multiple negative consequences, including performance impairment (Walsh et al., 2021; Castelli et al., 2022), as well as greater susceptibility to illness (Prather et al., 2015), injury (Huang and Ihm, 2021), depression (Perlman et al., 2006), psychological “burnout” (Gerber et al., 2018), and the “overtraining syndrome” (Meeusen et al., 2013). These consequences could negatively impact athletes for at least a season, and could contribute to termination of one’s athletic career (Holmes, 2018).

### Early morning training could be particularly problematic for high school and college athletes

To accommodate class schedules, two-a-day training necessitates early morning training for high school and college athletes. However, for multiple reasons, sleep loss associated with morning practices could be more problematic for high school and college athletes than for professional athletes. First, young athletes might have less opportunity to compensate for nighttime sleep loss with daytime napping, which can enhance performance (Botonis et al., 2021), because they often have classes that start at 8 a.m. and continue throughout the day. Second, young athletes are often unable to study until later in the night, after afternoon training and dinner. Third, among high school and college-age individuals there is a relatively high prevalence of a delayed circadian system and late bedtimes (night owls) (Crowley et al., 2007). Indeed, some evidence indicates that the average bedtime for college students is after midnight (Taylor and Bramoweth, 2010), and among extreme examples of circadian rhythm disorder delayed sleep phase type, individuals are unable to fall asleep until 2–3 a.m. or later.

### The problem could be intractable and more prevalent over time

It might be assumed that being a night owl, or even going to bed after midnight, is rare among athletes who train in the morning since an earlier sleep schedule (Eastman et al., 2005), morning light exposure (Kripke et al., 2007), and the exercise itself (Youngstedt et al., 2019) can help advance the circadian system to an earlier time. Studies show that athletes are able to go to bed and fall asleep earlier prior to early morning workouts compared with non-training days (Lastella et al., 2011; Sargent et al., 2014a; Sargent et al., 2014b). Moreover, self-selection probably also plays a role insofar as night owls are less willing and able to participate in sports requiring early morning training (Lastella et al., 2016). However, surveys suggest that a significant percentage of college athletes have delayed sleep schedules (Vitale et al., 2017). Moreover, evidence suggests that societal and environmental factors (e.g., nighttime light exposure) have led to a general tendency for people in the modern world to have more delayed body clocks (Roenneberg et al., 2012), a trend that could make this a more prevalent problem in the future. Finally, an expert panel recently concluded that there is still insufficient evidence that strategies to advance circadian time and sleep, such as morning light exposure, are effective for chronic management of individuals with delayed sleep phase syndrome (Auger et al., 2015) who face a chronic struggle to adapt to an earlier schedule. Likewise, research has indicated that early morning training is associated with less sleep (Sargent et al., 2014b) and going to bed earlier does not lead to adequate sleep duration among swimmers training in the morning (Sargent et al., 2014a).

It is hypothesized that shifting the early morning workout to a later time of day would be beneficial to these athletes. This hypothesis has not been tested.

### Potential barriers to such a change

There are several potential barriers and drawbacks that would need to be addressed before making such a change. These include the following.

#### Tradition and coaches’ attitudes

In an article titled “Morning Workouts are the Secret Pride of Swimmers,” author and collegiate swimmer Sarah Lloyd wrote “morning workouts are simultaneously the bane of and the reason for a swimmer’s existence (Lloyd, 2020).” Many coaches would be reluctant to alter workout times because of logistical difficulties (see below); not wanting to “pamper” athletes; and the “if it’s not broken, don’t fix it” rationale based on the storied history of swimmers who thrived on morning training (Lloyd, 2020).

The discipline required to, for example, regularly dive into cool water at 6 a.m., and other intangible factors such as the pride of doing so and the satisfaction associated with starting one’s day with a hard workout, could be irreplaceable. This is an empirical question that is worthy of investigation because the system of early morning training could indeed be “broken” for many young athletes. There could be Michael Phelps-level talent who would excel with later workout times but either never considered swimming because of the schedule; quit the sport; or failed to reach their potential because of an inability to shift their stubborn body clocks.

An analogy worth considering is a study of soldiers participating in basic combat training (Miller et al., 2012), another setting with a longstanding tradition of early morning physical training in young adults (Crowley et al., 2012). The study found that assigning half the soldiers to a later sleep schedule (11 p.m.–7 a.m.) compared with the control soldiers’ schedule (8:30 p.m. to 4:30 a.m.) resulted in significantly less fatigue, better marksmanship, and less total mood disturbance (Miller et al., 2012), which has been associated with better athletic performance in other studies (Morgan et al., 1987).

In summary, tradition and coaches’ attitudes are significant barriers to enacting the proposed changes in training schedule of two-a-day athletes. Nonetheless, definitive evidence supporting the benefits of such as change could persuade coaches to do this.

#### Logistics and safety

One approach would be to shift the start of the 6 a.m. workout to 9 or 10 p.m. for the entire team, which could be beneficial for most of the team. However, this could introduce safety challenges for workouts for some sports, such as running and cycling. There could also be safety concerns regarding commuting to and from the practice facilities, but perhaps not much different than those faced with 6 a.m. workouts for some of the year. The logistics of opening the workout facilities in the evening would be another challenge.

One concern might be that evening could disturb sleep, which would partly defeat the purpose of the proposed change. Nighttime athletic competition has resulted in disturbed sleep in some studies (Nédélec et al., 2019), but competition is associated with a far higher level of psychophysiological arousal than workouts. The available evidence indicates that even vigorous evening exercise ending 2-4 before bedtime does not disturb sleep for most individuals (Youngstedt et al., 1999; Buman et al., 2014; Vitale et al., 2017; Frimpong et al., 2001; Youngstedt et al., 2021), and with experience, it is possible that the minority of individuals with disturbed sleep after evening exercise can adapt to evening exercise without experiencing disturbed sleep.

Another option would be to shift the early morning workout to either the evening or later in the morning for some of the team, and keep the early morning workout for some of the team. However, this would introduce logistical challenges involving more hours of facility operation and more demands on the coaches’ time. Separating athletes could also be detrimental to team cohesion and the benefits of training with athletes of similar levels of performance.

In summary, logistical and safety issues are also significant barriers to shifting early morning workout times to later in the day. Whether these barriers can be overcome could vary for different teams, and might depend on the strength of research indicating advantages to making such changes.

#### Specificity of training time and performance

One of the rationales for morning swim training is that it will translate to better morning performance in preliminary trials, which are generally in the morning (e.g., 9 or 10 a.m. at the NCAA championship meet), and good performance in the trials is necessary to advance to the finals. Empirical support for this rationale is mixed.

There is a circadian rhythm in athletic performance with peak performance in the late afternoon to early evening and nadir of performance in the early morning (Youngstedt et al., 2019). However, Rae et al. found that swimmers who regularly trained only in the morning (average of 6 a.m.) swam faster in time trials at 6:30 a.m. compared with 6:30 p.m. (Rae et al., 2015). Nonetheless, this result could be partly explained by the fact that most of the morning swimmers were morning larks with a natural tendency to better morning performance.

Experimental studies have indicated that superior anaerobic exercise performance in the afternoon vs. morning can be significantly attenuated by anaerobic exercise training in the morning (6 a.m.) (Souissi et al., 2002; Chtourou and Souissi, 2012), and other research has indicated a greater improvement in anaerobic threshold in the morning and afternoon following training in the morning and afternoon, respectively (Hill et al., 1989). These results are consistent with compelling evidence that the time of day of exercise can shift the skeletal muscle clock (Martin and Esser, 2022) which could impact performance.

However, there are limitations of these data that make generalization to competition in elite athletes dubious. First, such evidence has not been shown in elite athletes. On the contrary, in a study of mostly collegiate swimmers, 200 m swim performance averaged almost 6 s slower at 3 a.m. and 6 a.m. compared with 8 p.m. and 11 p.m., despite regular morning training (Kline et al., 2007). Second, specificity of training effects have been shown for training at one time per day, and not for training twice per day. Third, specificity has been shown for performance measured within 30 min of training time. It is unclear, whether there is a similar advantage of training at 6 a.m. for performance at 9 or 10 a.m.

Nonetheless, the assumption persists that early morning workouts confer a competitive advantage for late morning competition Further research might be needed to confirm or dispel this assumption before coaches and athletes might be willing to forgo early morning workouts. Should more definitive evidence arise supporting this assumption, a subsequent important question is how long one must train in the morning to confer an advantage to morning performance. A study by Soussi et al. found that only 2 weeks of training twice per week in at 7–8 a.m. conferred improvement in Wingate and peak knee extension torque assessed at 7–8 a.m. (Souissi et al., 2002), and evidence indicates that a single bout of exercise can shift muscle clock gene expression in mice (Kemler et al., 2020). Thus, conceivably, athletes could experience a season of sufficient sleep, and benefit from a short period of early morning training close to major competitions.

## Discussion

The hypothesis could be tested in several ways.

### Epidemiological associations

Existing databases might have data regarding the athletes’ bedtimes, the extent to which they are night owls (e.g., the Horne Ostberg, Morningness-Eveningness questionnaire), their training schedules, performance across the season, and incidence of depressed mood, illness, and the overtraining syndrome. If not, these variables could be collected in the future to examine whether athletes with more delayed circadian timing are more likely to experience less sleep, worse performance and these other outcomes compared with those with more advanced circadian phase.

The question could be addressed best in individual sports for which performance can be objectively quantified, and is relatively unaffected by other factors such as climate or terrain (e.g., cross country). Swimming would be an ideal sport because of the standardized distances and relatively stable conditions. Performance could be quantified with respect to each individual’s personal records.

### Experimental testing

Experimental testing of the hypothesis would involve the logistical barriers listed above. One starting point could be to explore the hypothesis during the offseason, randomizing athletes to a shifted training schedule and others to the traditional 6 a.m. and 3 p.m. schedule. Another initial step could be to explore whether changing the workout schedule for the most extreme night owls has the proposed benefits. Possibly, a research team could work with a coach willing and able to test the proposed hypothesis in an entire team, or researchers could randomize a large group of participants to different workout schedules.

## Data Availability

The original contributions presented in the study are included in the article/Supplementary material, further inquiries can be directed to the corresponding author.
